# Neoadjuvant‐modified FOLFIRINOX vs nab‐paclitaxel plus gemcitabine for borderline resectable or locally advanced pancreatic cancer patients who achieved surgical resection

**DOI:** 10.1002/cam4.3075

**Published:** 2020-05-16

**Authors:** Adam R. Wolfe, Dhivya Prabhakar, Vedat O. Yildiz, Jordan M. Cloyd, Mary Dillhoff, Laith Abushahin, Dayssy Alexandra Diaz, Eric D. Miller, Wei Chen, Wendy L. Frankel, Anne Noonan, Terence M. Williams

**Affiliations:** ^1^ Department of Radiation Oncology Ohio State University James Comprehensive Cancer Center Columbus OH USA; ^2^ Department of Medical Oncology Ohio State University James Comprehensive Cancer Center Columbus OH USA; ^3^ Department of Biomedical Informatics Ohio State College of Medicine Columbus OH USA; ^4^ Department of Surgical Oncology Ohio State University James Comprehensive Cancer Center Columbus OH USA; ^5^ Department of Pathology Ohio State University James Comprehensive Cancer Center Columbus OH USA

**Keywords:** chemotherapy, FOLFIRINOX, nab‐paclitaxel, neoadjuvant, pancreatic cancer, radiation

## Abstract

We conducted an institutional study to compare the clinical and pathological efficacy between the neoadjuvant therapy (NAT)‐modified FOLFIRINOX (mFOLF) vs nanoparticle albumin–bound paclitaxel plus gemcitabine (nab‐P/G) for borderline resectable pancreatic cancer (BRPC) and locally advanced pancreatic cancer (LAPC) patients who completed resection. The study retrospectively enrolled patients with pathologically confirmed BRPC or LAPC from 2010 to 2018 at our institution. The survival rates were determined by the Kaplan‐Meier method and log‐rank test was used to test differences. Cox's proportional hazard model was used to assess survival with respect to covariates. Seventy‐two patients who completed at least two cycles of neoadjuvant chemotherapy and surgical resection were included, with 52 (72.2%) patients receiving mFOLF and 20 (27.8%) receiving nab‐P/G. Patients treated with mFOLF had statistically higher rates of RECIST 1.1 partial or complete response (16/52 vs 1/20, *P* = .028). Additionally, mFOLF patients had greater pathological tumor size reduction, fewer positive lymph nodes, and higher treatment response grade compared to the nab‐P/G patients (all *P* < .05). The median overall survival was 33.3 months vs 27.1 months (*P* = .105), and distant metastasis‒free survival (DMFS) was 21.3 months vs 14.6 months (*P* = .042) in the mFOLF vs nab‐P/G groups, respectively. On multivariate analysis, mFOLF (hazard ratio, 0.428; 95% confidence interval [CI], 0.186‐0.987) and abnormal postoperative CA 19‐9 (hazard ratio, 2.47; 95% CI, 1.06‐5.76) were associated with DMFS. Among patients with BRPC and LAPC who complete surgical resection, neoadjuvant mFOLF was associated with improved pathological and clinical outcomes compared with nab‐P/G.

## Graphical abstract

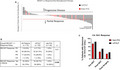

**Impact Statement**

Pancreatic ductal adenocarcinoma (PDAC) is a deadly disease with poor survival rates. For borderline resectable or unresectable disease, intensive multidrug chemotherapy regimens with either modified FOLFIRINOX (mFOLF) or nab‐paclitaxel plus gemcitabine are preferred. We retrospectively compared these two chemotherapy regimens in patients who completed resection and found the mFOLF group had better overall clinical and pathological response rates. Randomized clinical trials are needed, and this study provides valuable information in the interim.

## 1 INTRODUCTION

In the United States, there will be an estimated 56 770 new cases of pancreatic carcinoma (PC) and 45 750 estimated deaths in 2019.^1^ The 5‐year overall survival (OS) rate remains dismal at only 9%, and consequently, PC is projected to become the second leading cause of cancer‐related death by 2030.^2^ Forty percent of pancreatic ductal adenocarcinoma (PDAC) patients present with metastatic disease and of the remaining 60%, only 15% will ultimately achieve resection, the only curative treatment.^3^ PDAC patients with nonmetastatic and nonresectable tumors are typically divided between borderline resectable pancreatic cancer (BRPC) and locally advanced pancreatic cancer (LAPC). Converting patients from BRPC or LAPC to resectable status by means of aggressive neoadjuvant chemotherapy and/or radiation therapy (RT) is critical to improve outcomes for patients with nonmetastatic PDAC.

National Comprehensive Cancer Network (NCCN) defines BRPC based on the degree of tumor involvement of the major arteries and veins such as tumor abutment ≤ 180° of the superior mesenteric artery (SMA) or celiac axis and/or tumor contact > 180° of the superior mesenteric vein (SMV) or portal vein.^4^, ^5^ Current clinical guidelines for management of both BRPC and LAPC recommend neoadjuvant therapy (NAT) with multiagent chemotherapy followed by consideration of chemoradiation (CRT) or stereotactic body radiation therapy (SBRT) followed by surgical evaluation and resection, if feasible.^6^ Modified FOLFIRINOX (mFOLF) (5‐FU, leucovorin, irinotecan, and oxaliplatin) or nanoparticle albumin–bound paclitaxel (nab‐paclitaxel) plus gemcitabine (nab‐P/G) are the two most common chemotherapy regimens utilized in BRPC or LAPC. These regimens are now favored in the neoadjuvant setting based on two metastatic PDAC phase III trials that showed longer OS with the use of either mFOLF or nab‐P/G compared to gemcitabine alone.^7^, ^8^ In 2019, ASCO updated their guidelines to now recommend the use of adjuvant mFOLF as first‐line adjuvant chemotherapy for patients who undergo upfront resection on the basis of results from the PRODIGE 24‐ACCORD/CCTG PA‐6 phase III randomized trial that showed an OS improvement with the use of adjuvant mFOLF compared to gemcitabine.^9^, ^10^

To date. there are no reported prospective head‐to‐head comparison studies of mFOLF vs nab‐P/G in the adjuvant or neoadjuvant setting. There is now one ongoing randomized phase II trial comparing neoadjuvant and adjuvant mFOLF vs nab‐P/G for patients with resectable PDAC (NCT02562716). Given the slightly higher radiologic response rates with mFOLF vs nab‐P/G in the metastatic setting, we hypothesized mFOLF would result in greater radiographic, serum CA 19‐9, and pathological response rates compared to nab‐P/G in patients with BRPC and LAPC. To test this we performed a retrospective study in resected BRPC or LAPC patients after receiving either neoadjuvant mFOLF or nab‐P/G. We compared the tumor treatment effects based on changes in pre‐ and post‐NAT tumor size on imaging, changes in carbohydrate antigen 19‐9 (CA 19‐9), and treatment response grading on pathological reports between the two chemotherapy regimens, as well as clinical outcomes.

## 2 METHODS

### 2.1 Data sources and patient selection

We retrospectively identified all patients with a diagnosis of PDAC at the Ohio State University between September 2010 and May 2018 with nonmetastatic disease and initiated either mFOLF or nab‐P/G with curative intent. A total of 175 patients were identified, of whom 119 and 56 received either mFOLF or nab‐P/G, respectively. From this cohort, 52 (44%) and 20 (36%) achieved surgical resection without evidence of metastatic disease. The complete overview of all patients reviewed is displayed in Figure S1. We only included patients in this study with biopsy proven nonmetastatic BRPC or LAPC based on NCCN definitions (NCCN)^4^ and received a minimum of two cycles of either mFOLF or nab‐P/G. This was an IRB approved study from our institution (2014C0077).

### 2.2 Neoadjuvant treatments

The decision for neoadjuvant treatment was based on the decisions of the multidisciplinary pancreatic oncology team. The standard cycle of mFOLF consisted of oxaliplatin, 85 mg/m^2^; irinotecan, 165‐180 mg/m^2^; leucovorin followed by 2400 mg/m^2^ 46‐hour continuous infusion, all given every 2 weeks for 4‐8 cycles total. Patients receiving nab‐P/G were treated with a biweekly regimen of 1000 mg/m^2^ gemcitabine combined with nab‐paclitaxel, 125 mg/m^2^, administered on days 1 and 15 of a 28‐day cycle based on a previous published institutional study.^11^ Following completion of either chemotherapy, patients underwent restaging with CT or MRI. The decision on the use of radiation was made by the multidisciplinary team and was typically selected for patients with unresectable disease or concerns for margin positive resection. Radiation treatment volumes typically included the gross tumor volume (GTV) and any grossly involved nodes on imaging with a 1.0‐1.5 cm margin using either 3D‐conformal radiation (3DCRT) or intensity modulated radiation therapy (IMRT). Concurrent chemotherapy with RT was weekly gemcitabine at 1,000 mg/m^2^ or 5‐FU depending on provider's preference and response to prior chemotherapy.

### 2.3 Tumor characteristics and response

CA19‐9 (U/mL) was recorded at diagnosis prior to NAT, post‐NAT, and postsurgery at the closest time points. Tumor size (cm), location, and baseline clinical stage were evaluated using CT of the chest, abdomen, pelvis, and MRI when available. Clinical staging was documented according to the American Joint Committee on Cancer (AJCC) Cancer Staging Manual, 8th Edition (2018). The most recent staging system was used for all patients as the time period for this study spanned editions 7 and 8. Clinical response was determined based on the response evaluation criteria in solid tumors (RECIST 1.1) criteria.^12^

### 2.4 Pathological outcomes

Pathology reports from surgical resections were reviewed in the electronic medical record for each patient and tumor size, T‐stage, N‐stage, total number of lymph nodes positive and removed, grading pathologic treatment response, as well as presence of lymphovascular invasion (LVI), and/or perineural invasion (PNI) were recorded. Pathological assessment of tumor response to neoadjuvant therapy is performed using the modified Ryan scheme^13^ as recommended by AJCC Cancer Staging Manual 8th Ed. and College of American Pathologists. Tumor regression score (TRS) ranges from 0 to 3, and is defined as follows: 0 = No viable cancer cells (complete response), 1 = Single cells or rare small groups of cancer cells (near complete response), 2 = Residual cancer with evident tumor regression, but more than single cells or rare small groups of cancer cells (partial response), 3 = Extensive residual cancer with no evident tumor regression (poor or no response).

### 2.5 Survival outcomes

OS was defined as duration from diagnosis to date of death from any cause in months. Local and distant recurrences were documented based on postsurgical review of CT images and follow‐up notes. Progression‐free survival (PFS) in months was determined from the date of diagnosis to either local or distant tumor recurrence, or death. Distant metastasis‐free survival (DMFS) was determined from the date of diagnosis to date of distant recurrence in months or death. Local recurrence‐free survival (LRFS) was defined from the date of surgery to date of any recurrence in the surgical resection bed or draining lymph nodes or death. Patients were not censored following recurrence and could develop a local or distant event following the initial distant or local event or vice versa.

### 2.6 Statistics

Prior to analysis, the data were examined for outliers; no extreme values were found. For the group comparisons, two‐sample *t* test or Mann‐Whitney *U* test was used for the continuous variables and chi‐squared or Fisher's exact test was used for the categorical variables as appropriate. Group differences of time to event outcomes were visually inspected using Kaplan‐Meier curves. The log‐rank test was used to assess the statistical significance between groups. Cox's proportional hazard model was used to assess survival with respect to covariates (age, PNI, LVSI etc) simultaneously. The significance level was set at *α* ≤ 0.05. The data were analyzed using Statistical Analysis Software, version 9.4 (SAS Institute Inc) and SPSS (Version 25.0. Armonk, NY: IBM Corp).

## 3 RESULTS

### 3.1 Patient demographics

Seventy‐two patients met the inclusion criteria for this analysis with pathologically confirmed diagnosis of nonmetastatic PDAC who were deemed borderline resectable or unresectable after multidisciplinary tumor board review. Patients were treated with either neoadjuvant mFOLF (n = 52, 72.2%) or nab‐P/G (n = 20, 27.8%). Patients in the mFOLF group were younger, had better performance status, and were less likely to have a diagnosis of diabetes compared to the nab‐P/G group (median age; 61.4 years vs 71.6 years *P* < .001, ECOG < 1; 55.8% vs 20% *P* = .002, diabetes diagnosis; 19.2% vs 55% *P* = .008 for mFOLF vs nab‐P/G, respectively). There were no statistical differences in race, gender, BMI, or smoking status (Table 1).

**Table 1:** Demographic and clinical characteristics of the study cohort pre‐NAT

Variable	All patients (n = 72)	mFOLF (n = 52)	Nab‐P/G (n = 20)	*P*
Age				**<.001** ^a^
Median (range)	66 (32, 86)	62.5 (32, 75)	72.0 (50, 86)	
Sex				.59^b^
Male	29 (40.3%)	21 (40.4%)	8 (40.0%)	
Female	43 (59.7%)	31 (59.6%)	12 (60.0%)	
ECOG				**.002** ^b^
1	33 (45.8%)	29 (55.8%)	4 (20.0%)	
2	35 (48.6%)	19 (36.5%)	16 (80.0%)	
3	4 (5.6%)	4 (7.7%)	0 (23.7)	
Race				.24^b^
White	65 (90.3%)	47 (90.4%)	47 (90.0%)	
Black	4 (5.6%)	2 (3.8%)	2 (10.0%)	
Asian	3 (4.2%)	3 (5.8%)	0 (0.0%)	
BMI (kg/m^2^)				.43^a^
Median (range)	24.6 (18.3, 45.2)	24.6 (18.3, 45.2)	26.15 (19.0, 44.4)	
Smoking history				.13^b^
Never	41 (56.9%)	27 (51.9%)	14 (70.0%)	
Former/Current	31 (43.1%)	25 (48.1%)	6 (30.0%)	
Diabetes				**.008** ^b^
No	51 (70.8%)	42 (80.8%)	9 (45.0%)	
Yes	21 (29.2%)	10 (19.2%)	11 (55.0%)	
Tumor location				.53^b^
Head/Neck	48 (66.7%)	35 (67.3%)	13 (65.0%)	
Body/Tail	24 (33.3%)	17 (32.7%)	17 (35.0%)	
Resectability^d^				.37^b^
Borderline	50 (69.4%)	35 (67.3%)	15 (75.0%)	
Unresectable	22 (30.6%)	17 (32.7%)	5 (25.0%)	
Maximal tumor dimension on imaging (cm)				.072^a^
Median (range)	3.3 (1.6, 6.4)	3.15 (1.6, 5.8)	3.79 (2.7, 6.4)	
Clinical T‐stage^c^				.49^b^
1	1 (66.7%)	1 (1.9%)	0 (67.1%)	
2	38 (52.8%)	26 (50.0%)	12 (60.0%)	
3	11 (15.3%)	7 (13.5%)	4 (20.0%)	
4	22 (30.6%)	18 (34.6%)	4 (20.0%)	
Clinical N‐stage^c^				.56^b^
N0	53 (73.6%)	38 (73.1%)	15 (75.0%)	
N1‐2	19 (26.4%)	14 (26.9%)	5 (25.0%)	
Arterial involvement				.31^b^
No	31 (43.1%)	21 (40.4%)	10 (50.0%)	
Yes	41 (56.9%	31 (59.6%)	10 (50.0%)	
Venous involvement				.14^b^
No	14 (19.4%)	8 (15.4%)	6 (30.0%)	
Yes	58 (80.6%)	44 (84.6%)	14 (70.0%)	

*P* values < .05 met our threshold for significance and are labeled in bold.

Abbreviations: BMI, body mass index; ECOG, Eastern Cooperative Group; mFOLF, modified FOLFIRINOX; Nab‐P/G, nab‐paclitaxel plus gemcitabine; NAT, neoadjuvant therapy.

^a^ Mann‐Whitney *U* (Wilcoxon rank‐sum) test.

^b^ Fisher's exact test.

^c^ American Joint Committee on Cancer (AJCC) 8th edition.

^d^ NCCN Criteria Defining Resectable Status Version 1.2019.

### 3.2 Tumor characteristics

We found no statistical differences comparing the two chemotherapy groups with regard to pancreatic tumor location (65% vs 67% head or neck location), resectability (67% vs 75% borderline resectable), clinical tumor size (median 3.15 cm vs 3.79 cm, *P* = .072), T‐stage, N‐stage, clinical stage, artery, or venous involvement (Table 1).

### 3.3 Neoadjuvant treatment characteristics

The median neoadjuvant chemotherapy cycles was 3.0 for both the mFOLF (range 2‐8) and nab‐P/G (range 2‐6) groups, respectively. The rate of adjuvant chemotherapy use was not statistically different between the two groups (67.3% vs 50%) (*P* = .18), and 52.7% of all patients completed at least 6 months of neoadjuvant and adjuvant chemotherapy (59.6% vs 35.0% for mFOLF and nab‐P/G, respectively *P* = .06). RT was delivered to 34 (65.4%) patients in the mFOLF group and 11 (55%) patients in the nab‐P/G group (*P* = .29). The median radiation dose was similar between the two groups (36 Gy mFOLF vs 39 Gy nab‐P/G). There were no significant differences in radiation dose, dose per fraction, or type of fractionation pattern between mFOLF and nab‐P/G. The time from diagnosis to surgery did not differ between the two chemotherapy groups (mean 6.38 vs 6.41 months, *P* = .95) (Table 2).

**Table 2:** Treatment characteristics of the study cohort

Variable	All patients (n = 72)	mFOLF (n = 52)	Nab‐P/G (n = 20)	*P*
Induction CT cycles				.73^a^
Median (range)	3.0 (2, 8)	3.0 (2, 8)	3.0 (2, 6)	
Adjuvant CT delivered?				.18^b^
Yes	45 (62.5%)	35 (67.3%)	10 (50.0%)	
Adjuvant CT cycles				.29^a^
Median (range)	1 (0, 13)	2.0 (0, 13)	1.0 (0, 8)	
Completed total 6 mo of neoadjuvant ± adjuvant CT?				.06^b^
Yes	38 (52.7%)	31 (59.6%)	7 (35.0%)	
Received neoadjuvant RT?				.29^b^
Yes	45 (62.5%)	34 (65.4%)	11 (55.0%)	
Total radiation dose (Gy)				.71^a^
Median (range)	36 (30‐54)	36 (33‐54)	39 (30‐50.4)	
Concurrent CT with RT				.67^b^
5‐FU/capecitabine	5 (5.9%)	3 (5.8%)	2 (10.0%)	
Gemcitabine	37 (51.4%)	29 (55.8%)	8 (40.0%)	
None	3 (4.2%)	2 (3.8%)	1 (5.0%)	
Type of Surgery				.15^b^
Whipple	41 (56.9%)	29 (55.8%)	12 (60.0%)	
Distal pancreatectomy	19 (26.4%)	12 (23.1%)	7 (35.0%)	
Appleby	3 (4.2%)	2 (3.8%)	1 (5.0%)	
Total pancreatectomy	8 (11.1%)	8 (15.4%)	0 (0.0%)	
Time from diagnosis to surgery (months)				.95^a^
Median (range)	6.4 (2.1,14.6)	6.4 (3.1, 12.0)	6.3 (2.1, 14.7)	

Abbreviations: 5‐FU: 5‐fluorouracil; CT, chemotherapy; mFOLF, modified FOLFIRINOX; Nab‐P/G, nab‐paclitaxel plus gemcitabine; RT, radiation therapy.

^a^ Mann‐Whitney *U* (Wilcoxon rank‐sum) test.

^b^ Fisher's exact test.

### 3.4 Clinical imaging and biomarker response rates

**Figure 1:**
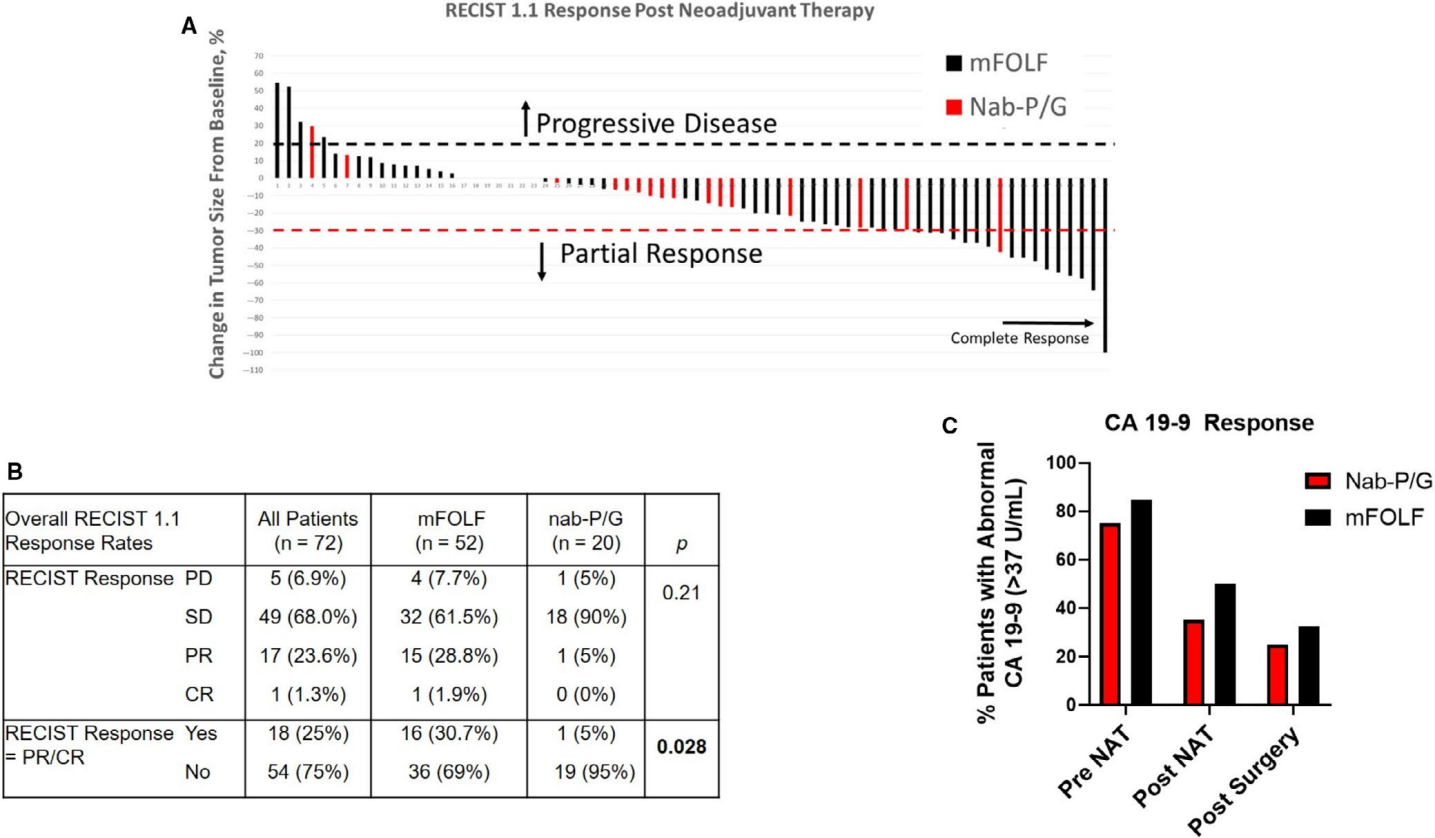
Clinical response to neoadjuvant chemotherapy. A, Waterfall plot of all 72 patients based on change in tumor size based on imaging pre‐ vs post‐NAT. The dashed line above the *x*‐axis represents 20% increase in sum of target lesions from baseline (progressive disease (PD)), and dashed line below the *x*‐axis represents 30% decrease in sum of target lesions from baseline (partial response (PR)). In this cohort, the majority of patients had stable disease based on RECIST 1.1 criteria. B, Using RECIST 1.1 criteria, patients were classified based on clinical response. PD = progressive disease ≥ 20% increase in size, PR = partial response ≥ 30% decrease in size, SD = stable disease, neither PD or PR, and CR = complete response disappearance of lesion. C, Median CA 19‐9 (U/mL) were recorded for all patients pre‐NAT, post‐NAT, and postsurgery within 1‐month of each time point

Tumor size dimensions (cm) based on CT imaging were recorded at baseline before the delivery of NAT and after NAT just prior to surgery. The majority of patients following neoadjuvant mFOLF (61.5%) or nab‐P/G (85%) had stable disease (SD) (Figure 1A). There was only one patient in the entire cohort who received mFOLF that achieved a clinical complete response (cCR). Patients receiving mFOLF group had statistical higher RECIST 1.1 response rates of PR or CR (n = 16, 30.7%) vs the nab‐P/G group (n = 1, 5%, *P* = .028) (Figure 1B). The average reduction in tumor size was 17% vs 12% in the mFOLF (3.31 cm pre‐NAT, 2.76 post‐NAT) vs nab‐P/G (3.79 cm pre‐NAT, 3.35 cm post‐NAT), respectively. For those with available CA 19‐9 data, the level of serum CA 19‐9 at diagnosis was 75% higher in the mFOLF group (median = 259.2 U/mL) compared to the nab‐P/G group (median = 148.15 U/mL), although the decline in CA 19‐9 was similar in both groups, 84% in the mFOLF group (median 259.25 pre‐NAT, 41.4 post‐NAT) vs 80% in the nab‐P/G group (median 148.15 pre‐NAT, 29.05 post‐NAT). The percentage of patients with abnormal CA 19‐9 levels were slightly higher in the mFOLF group at all three timepoints (Figure 1C).

### 3.5 Pathological response outcomes

As shown in Table 3, the R0 resection rate between the two groups was similar (73.1% vs 75%, *P* = 1.0). The pathological factors that were significantly different between the groups were pathological tumor size, number of positive lymph nodes, and treatment response grade. The median tumor size was 2.34 cm vs 3.16 cm in the mFOLF vs nab‐P/G groups, respectively (*P* = .03). The number of pathological positive lymph nodes was 1.33 vs 2.90 in the mFOLF vs nab‐P/G groups, respectively (*P* = .019). There were two patients (3.9%) in the mFOLF group who achieved a pathological complete response vs none in the nab‐P/G group. Ten (19.2%) patients in the mFOLF group were graded by the pathologists as either having a complete (grade 0) or extensive TRS (grade 1) compared to no patients in the nab‐P/G group (*P* = .05). Other pathological factors analyzed were not significantly different between the groups including perineural invasion, lymphovascular invasion, number of lymph nodes removed, and tumor grade (all *P* > .05). To determine if age or ECOG performance status were biasing these pathological findings, we analyzed the two pathological findings in the mFOLF cohort that were found to be significantly different, specifically the number of positive lymph nodes (LNs) and TRS, based on age and ECOG performance status (PS). First, in terms of number of LNs positive, we found there was a nonsignificant inverse correlation between number of positive lymph nodes and age (Pearson = −0.113, *P* = .43). Second, in terms of ECOG PS status, there was a nonsignificant trend toward worse ECOG PS and a higher number of LNs positive (ECOG 0 = 0.82, ECOG 1 = 1.84, ECOG 2 = 2.5, *P *= .08) Next, we looked at age and ECOG PS with regards to pathological treatment response. First, with regard to age, there was no differences in age and achieving a complete (Grade 0) or near complete response (Grade 1) vs partial response (Grade 2) or no response (Grade 3). Mean age for both groups was roughly 61 years (*P* = .8). Second, for ECOG PS and treatment response grade, there was a relatively even distribution between ECOG PS 0‐2 and treatment response grade 0‐1 vs 2‐3, indicating PS was not correlated with alterations in treatment response.

**Table 3:** Pathological characteristics of the study cohort

Pathological variable	mFOLF (n = 52)	Nab‐P/G (n = 20)	*P*
R0 resection			1.00^b^
Yes	38 (73.1%)	15 (75.0%)	
Tumor size (cm)			**.03** ^a^
Mean (range)	2.34 (0, 5.8)	3.16 (1.4, 6.1)	
Tumor histological grade			.14^b^
1‐2	34 (65.4%)	17(85.0%)	
3	18 (34.6%)	3 (15.0%)	
PNI			.53^b^
Positive	40 (76.9%)	17 (85.0%)	
LVSI			.11^b^
Positive	25 (48.1%)	14 (70.0%)	
pT‐Stage^c^			.35^b^
1‐2	42 (80.8%)	14 (70.0%)	
3‐4	10 (19.2%)	6 (30.0%)	
pN‐Stage^c^			.72^b^
0	24 (46.2%)	8 (40.0%)	
1‐2	28 (53.8%)	12 (60.0%)	
Lymph nodes positive			**.02** ^a^
Mean (range)	1.3 (0, 7)	2.9 (0, 11)	
Lymph nodes examined			
Mean (range)	22.7 (11, 55)	24.1 (12, 38)	.54^a^
Lymph node ratio (%)			0.08^a^
Mean (range)	6 (0,46)	11 (0, 55)	
TRS^d^			**.05** ^b^
0‐1	10 (19.2%)	0 (0.0%)	
2‐3	42 (80.8%)	20 (100%)	

P values < .05 met our threshold for significance and are labeled in bold. Abbreviations: LVSI, lymphovascular invasion; mFOLF, modified FOLFIRINOX; Nab‐P/G, nab‐paclitaxel plus gemcitabine; PNI, perineural invasion.

^a^ Student's *t* test.

^b^ Fisher's exact test.

^c^ American Joint Committee on Cancer (AJCC) 8th edition.

^d^ TRS—Tumor Regression Score—Grade 0 = complete response, 1 = near complete response, 2 = partial response, 3 = poor or no response.

### 3.6 Survival outcomes

**Figure 2:**
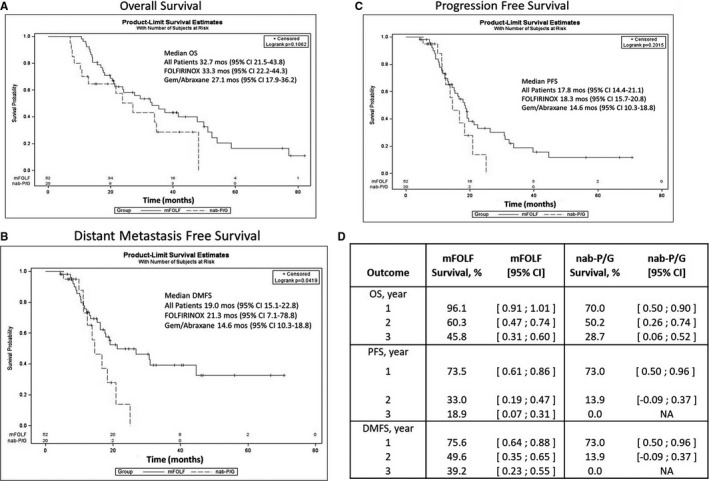
A, Overall survival (OS) B, Distant metastasis‒free survival (DMFS) and C, Progresion‐free survival (PFS) stratified based on the NAT chemotherapy group. D, 1‐, 2‐, and 3‐ year OS, PFS, and DMFS by the chemotherapy group

The median follow‐up was 19.0 and 17.3 months in the mFOLF and nab‐P/G groups, respectively. There was no significant difference in OS between the two chemotherapy groups, with the median OS of 33.3 months in the mFOLF group vs 27.1 months in the nab‐P/G group, *P* = .105 (Figure 2A). Interestingly, the mFOLF group had significantly longer DMFS vs nab‐P/G patients (21.3 months vs 14.6 months, *P* = .042) (Figure 2B). PFS was not significant between the two groups (Figure 2C). The 1‐, 2‐, and 3‐year OS, PFS, and DMFS were all higher in the mFOLF group compared to the nab‐P/G group (Figure 2D). We performed univariate analysis of variables associated with OS and DMFS (Supplementary Table 1), and found that older age, abnormal postoperative CA 19‐9, R1 resection, larger tumor size, higher pathologic T‐stage, and higher posttreatment response grade were associated with worse OS and were included in the multivariate analysis (*P* < .10). Variables associated with DMFS and included in the multivariate analysis were: node positivity, nab‐P/G treatment, abnormal pretreatment/posttreatment/postoperative CA 19‐9, R1 resection, increased tumor size, higher tumor grade, higher pathologic T‐stage, and worse treatment response (*P* < .1). After multivariate adjustment, R1 resection and pathological T‐stage remained significant for OS, and the variables that predicted for improved DMFS were the use of mFOLF and normal postsurgical CA 19‐9 levels (Table 4).

**Table 4:** Multivariate hazard ratios for overall survival and distant metastasis‐free survival*

	OS hazard ratio (95% CI)	*P*	DMFS hazard ratio (95% CI)	*P*
Chemo group				
nab‐P/G	—	—	1.0	—
mFOLF	—	—	0.428 (0.19 to 0.99)	**.046**
Age (y)	1.02 (0.98 to 1.07)	.33	—	—
Pathological tumor size (cm)	1.19 (0.71 to 1.98)	.50	0.95 (0.70 to 1.28)	.73
Path T‐stage^a^				
4	1.0	—	—	—
3	0.14 (0.02 to 0.99)	**.049**	—	—
2	0.15 (0.04 to 0.60)	**.007**	—	—
Clinical N‐stage^a^				
1‐2	—	—	1.0	—
0	—	—	0.48 (0.21 to 1.08)	.08
Postoperative CA 19‐9				
Normal (0‐37 U/mL)	1.0	—	1.0	—
Abnormal (>37 U/mL)	2.13 (0.99‐4.60)	.051	2.47 (1.06 to 5.76)	**.035**
Preoperative CA 19‐9				
Normal (0‐37 U/mL)	—	—	1.0	—
Abnormal (>37 U/mL)	—	—	0.70 (0.27 to 1.82)	.47
Pre‐NAT CA 19‐9				
Normal (0‐37 U/mL)	—	—	1.0	—
Abnormal (>37 U/mL)	—	—	2.07 (0.63 to 6.86)	.23
Histological tumor grade	—	—	1.0	—
1‐2	—	—	1.56 (0.73 to 3.33)	.25
3				
Resection margin status				
R1	1.0	—	1.0	—
R0	0.33 (0.17‐0.66)	**.002**	0.47 (0.21 to 1.04)	.06
TRS				
2‐3	1.0	—	1.0	—
0‐1	0.66 (0.18‐2.45)	0.53	0.41 (0.10 to 1.63)	.20

P values < .05 met our threshold for significance and are labeled in bold. Abbreviations: CI, confidence interval; DMFS, distant metastasis‒free survival; NAT, neoadjuvant therapy; OS, overall survival; TRS, tumor regression score.

^a^ American Joint Committee on Cancer (AJCC) 8th edition.

* Variables for multivariate analysis were selected based on significant variables (*P* < .10) on univariate analysis (Table S1).

### 3.7 Radiation therapy impact

We next explored whether neoadjuvant radiation improved pathological response or survival outcomes. We split the cohort into two groups, chemotherapy alone (n = 27) vs chemotherapy plus chemoRT (n = 45). The chemoRT group had a longer interval time from diagnosis to surgery (mean 7.31 months vs 4.85 months, *P* < .001). ChemoRT was associated with decreased pathologic nodal involvement and increased pathological treatment response. Pathological nodal stage, number of node positives, and lymph node ratio were all lower in the chemoRT group (*P* < .05). Poor treatment response (grade 3) was more likely in patients who received chemotherapy alone compared to chemoRT (44.4% vs 17.8%, *P* = .028) (Table S2). The chemoRT group had nearly double the median LRFS compared to the chemo alone group (median 23.6 months vs 11.5 months, *P* = .112), but there was not a significant difference in OS in the two groups (Figure S2).

## 4 DISCUSSION

In this retrospective single institutional study, we found the use of neoadjuvant mFOLF in patients with initially either BRPC or LAPC who underwent successful surgical resection had increased pathological nodal downstaging and pathological treatment response compared to nab‐P/G. Furthermore, mFOLF was associated with longer DMFS vs gem‐P/G on multivariate analysis. Currently, both mFOLF and nab‐P/G are favored over gemcitabine in the metastatic setting based on the PRODIGE 4 ACCORD 11 and MPACT trials which showed improved outcomes with either mFOLF or nab‐P/G vs gemcitabine alone. mFOLF compared to gemcitabine improved OS in metastatic PC patients from 6.8 to 11.1 months,^8^ and nab‐P/G improved OS from 6.7 to 8.5 months.^7^ A review by Ghosn et al of these two metastatic trials showed mFOLF had higher rates of overall response (31.6% vs 21%), disease control (70.2% vs 48%), and 1‐year OS (48.4% vs 35%). Toxicity appeared to be worse in mFOLF in terms of higher rates of neutropenia (45.7% vs 38%), febrile neutropenia (5.4% vs 3%), and diarrhea (12.7% vs 6%). Nab‐P/G had higher rates of thrombocytopenia (13% vs 9.1%), anemia (13% vs 7.8%), alopecia (50% vs 11.2%), and toxic death (4% vs 0.6%).^14^

There are now two randomized phase III trials either completed or closed that compared either adjuvant mFOLF or nab‐P/G vs gemcitabine alone for resected PDAC patients. The PRODIGE 24‐ACCORD/CCTG PA‐6 study found adjuvant mFOLF had an improved median OS of 54.4 months compared to 35 months in the gemcitabine group (HR = 0.64, *P* = .003).^9^ The APACT trial did not show improvement in the primary endpoint, independently assessed PFS, for adjuvant nab‐P/G vs gemcitabine alone (NCT01964430); however, interim OS (427 events) was improved, with 40.5 months in the nab‐P/G group vs 36.2 months in the gemcitabine group (HR, 0.82; 95% CI, 0.680‐0.996; nominal *P* = .045).^15^ A similar retrospective analysis did report improved resection rates, PFS, and OS with mFOLF vs nab‐P/G^16^; however, conclusions about efficacy in this study are limited by the finding that younger and better performance status patients were more likely to receive mFOLF. ASCO guidelines recommend the use of multiagent chemotherapy for patients with good performance status, but the choice of which chemotherapy regimen is left up to the discretion of the physician.^6^

In the neoadjuvant setting, multiple retrospective and prospective studies have now been reported on mFOLF clinical outcomes. A meta‐analysis of 13 studies and 689 patients with LAPC reported a median OS between 23.1‐42.3 months and found 25.9% who were treated with mFOLF underwent successful resection.^17^ Intergroup Alliance trial A021101, was a phase II trial of BRPC in which patients received mFOLF for 4 cycles followed by concurrent capecitabine and RT to 50.4 Gy. In this trial, 68% underwent surgery and, of the resected patients, the 12 and 18 month OS was 73% and 43%, respectively.^18^ Murphy and colleagues’ phase II neoadjuvant BRPC trial reported a regimen of 8 cycles of mFOLF followed by either a short course RT (5 fractions, 25 Gy) or long‐course RT (50.4 Gy) which resulted in an R0 resection rate of 65% and, of the resected patients, a 2‐year PFS and OS of 43% and 56%, respectively.^19^ Another phase II neoadjuvant LAPC trial from this group followed the same mFOLF + RT regimen but also added the angiotensin II receptor blocker, losartan, which resulted in an R0 resection rate of 69% and median OS of 33 months, well above historical standards for LAPC.^20^ Prospective data on outcomes with neoadjuvant nab‐P/G have not been reported at the time of this manuscript submission.

Among patients undergoing NAT, the optimal means of measuring response to treatment is poorly understood. These are important because they guide treatment decision making and are prognostic. There are three current ways to assess NAT response; (a) biochemical, (b) radiographic, and (c) pathologic. Biochemical response can be measured with CA 19‐9, a circulating tumor‐associated antigen used in the setting of a new PDAC diagnosis or following resection to monitor disease recurrence. Postoperative values > 90 U/mL predicted for worse OS in the phase III RTOG 9704 trial.^21^ In the neoadjuvant setting, Tzeng et al found for patients treated with NAT, normalization of CA 19‐9 following chemotherapy was associated with longer median OS for resected and nonresected patients.^22^ Boone et al retrospectively analyzed 78 patients treated with NAT and found a pathological response rate of 29% for patients with a CA 19‐9 decline of > 90% vs 0% in those patients with < 90% CA 19‐9 decline.^23^ Finally Mattiucci et al analyzed 404 patients from eight institutions and found presurgical CA 19‐9 levels independently predicted for worse OS on multivariate analysis.^24^ In our study, we found postoperative CA 19‐9 predicted for worse OS on univariate analysis and DMFS on both univariate and multivariate analysis. Importantly, abnormal CA 19‐9 values both pre‐ and post‐NAT did not predict for OS or DMFS on multivariate analysis. Patients in the mFOLF group had almost twice the level of CA 19‐9 at baseline compared to the nab‐P/G group, but had similar postsurgical CA 19‐9 normalization.

Radiographic response to NAT is not clearly associated with outcomes. Ferrone et al examined 40 patients after neoadjuvant mFOLF and RT who completed surgical resection. Nineteen were deemed radiographically unresectable, and yet there was a 92% R0 resection rate.^25^ Katz et al concluded from their retrospective analysis of pre‐ and post‐NAT CT images of BRPC, radiographic downstaging was rare and not predictive of treatment outcomes. There report found only one (0.8%) patient who had disease down‐staged to resectable following NAT, although 85 (66%) underwent surgery.^26^ In this study, we found patients in the mFOLF group to have higher rates of RECIST 1.1 response of PR or CR compared to nab‐P/G (*P* = .03) (Figure 1). Similar to prior reports, the majority of patients in this cohort had stable disease following NAT. Finally, pathological response grading is not well established. There are currently several grading criteria including the College of American Pathologists (CAP), Evans, and MDACC grading systems. Pathological complete response (pCR) are exceedingly rare after NAT for PC but for patients that do achieve either a pCR or < 5% residual viable tumor cells, they have over twice the median OS compared to those that did not (73.4 months vs 32.2 months, *P* < .001).^27^ In this cohort, mFOLF treated patients that had higher rates of TRS complete (Grade 0) or extensive (Grade 1) compared to the nab‐P/G patients. There were only 2 (2.8%, both in the mFOLF group) patients who achieved a pCR, similar to prior findings.^28^ This highlights the critical need for novel treatments and improved biomarkers.

Another unknown area in the NAT setting is the role for radiation. There has been mixed results for survival benefit in the LAPC setting for radiation (GITSG^29^ and ECOG 4201^30^ showed an OS benefit and FFCD‐SFRO^31^ and LAP07^32^ found no OS benefit for radiation). Local control is clearly important in PDAC based on autopsy results showing approximately 30% of patients died directly of complications from local failure.^33^ The LAP‐07 trial is often cited to argue against the standard use of chemoRT in the LAPC setting due to the lack of an OS benefit, although there was significantly less local‐regional tumor progression compared to the chemotherapy alone arm (32% vs 46%, *P* = .03).^32^ Retrospective studies of chemoRT in the neoadjuvant setting have similarly shown improved locoregional control without an effect on OS.^34^, ^35^ In the current era, patients are now treated with several months of chemotherapy and then restaged before proceeding to chemoRT, a process which selects out for patients with early distant metastasis and who may be less likely to achieve potential clinical benefit from local control afforded by radiation. We found in our study chemoRT improved pathological response, nodal downstaging, and local‐regional recurrence‐free survival, but similar to other studies, OS was not improved.

Currently, there is an active phase II trial from SWOG (S1505) that is enrolling patients with resectable disease to receive either neoadjuvant and adjuvant mFOLF or neoadjuvant nab‐P/G and adjuvant nab‐P/G with a 2‐year OS “pick the winner” design (NCT02562716). We anticipate the results of this study will help elucidate the choice of chemotherapy for resectable PDAC and could potentially be extrapolated in BRPC or LAPC settings.

As in all retrospective studies, this study has several limitations. The biggest limitation is the lack of an assessment of how many patients did not undergo surgery who were initially treated with mFOLF or nab‐P/G, since only patients who had surgery were included. As such, we did not assess the important question of which regimen is most associated with patients undergoing surgery after completion of preoperative therapy. Of note, in our institution's complete database of patients treated with neoadjuvant chemotherapy ± radiation, more patients treated with mFOLF achieved surgical resection (44% vs 36%, Figure S1). This would further support the notion mFOLF has better biological response. Another important limitation was that the choice of mFOLF or nab‐P/G was not randomized. As noted in our patient characteristics, patients receiving mFOLF were younger and had better performance status, which temper any conclusions that can be made on survival outcomes. Additionally there was a trend for patients in the mFOLF group to receive more adjuvant cycles of chemotherapy which could potentially account for better metastasis‐free survival. Finally, the small sample size may limit the power of the study to detect differences between the two groups. However, the focus of this study was not clinical outcomes or the surgical resection rates, which are indeed heavily biased on patient characteristics such as age or performance status, but specifically this study focused on the biological tumor response based mainly on the pathology report. The pathologist on these cases were agnostic to the patient's age or performance status and therefore the pathological response in terms of lymph node downstaging and neoadjuvant treatment response scores would be an unbiased metric to evaluate the two chemotherapy regimens. In order to determine if age or ECOG PS could bias the pathological outcomes favoring mFOLF, we ran a sub analysis of the two pathological findings that were significantly different between the two chemotherapy regimens, specifically the number of positive lymph nodes (LNs) and TRS. We found no significant findings for either age or ECOG PS on TRS or pathological number of positive lymph nodes. Taken together, we feel that both age and ECOG performance status are not biasing the biological response rates found on the pathology reports between the two chemotherapy regimens. A larger cohort of patients would better assist in answering this question, as we are underpowered to perform these subgroup analyses due to only have 10 patients with pathologic response grade of 0‐1 in the mFOLF group. We report clinical outcomes as a secondary analysis but not as the major focus. Importantly, although the two arms were unbalanced in terms of age and PS, there were no statistical differences between the groups in terms of number of chemotherapy cycles (both median of 3), radiation delivered, or adjuvant chemotherapy (Table 2).

## 5 CONCLUSIONS

In conclusion, in our institutional cohort of resected PDAC patients, neoadjuvant mFOLF improved pathological nodal downstaging and treatment response in patients with BRPC or LAPC compared to nab‐P/G. We also observed an improvement in DMFS for patients receiving mFOLF. Larger sample sizes and prospective studies are needed to validate these results and optimize the chemotherapy regimen in BRPC and LAPC patients.

## AUTHOR CONTRIBUTION

All listed authors have made substantial contributions to conception and design, or acquisition of data, or analysis and interpretation of data.

## CONFLICT OF INTEREST/FINANCIAL DISCLOSURES

All authors have no conflicts of interest or competing financial interests to disclose.

## Supporting information

Fig S1

Fig S2

Table S1

Table S2

## Data Availability

The data that support the findings of this study are available on request from the corresponding author. The data are not publicly available due to privacy or ethical restrictions.
