# Supplementary figures and images for: Neoadjuvant‐modified FOLFIRINOX vs nab‐paclitaxel plus gemcitabine for borderline resectable or locally advanced pancreatic cancer patients who achieved surgical resection
Source: Cancer Med. 2020 May 16;9(13):4711–23. doi: 10.1002/cam4.3075 (PMC7333854; doi:10.1002/cam4.3075)

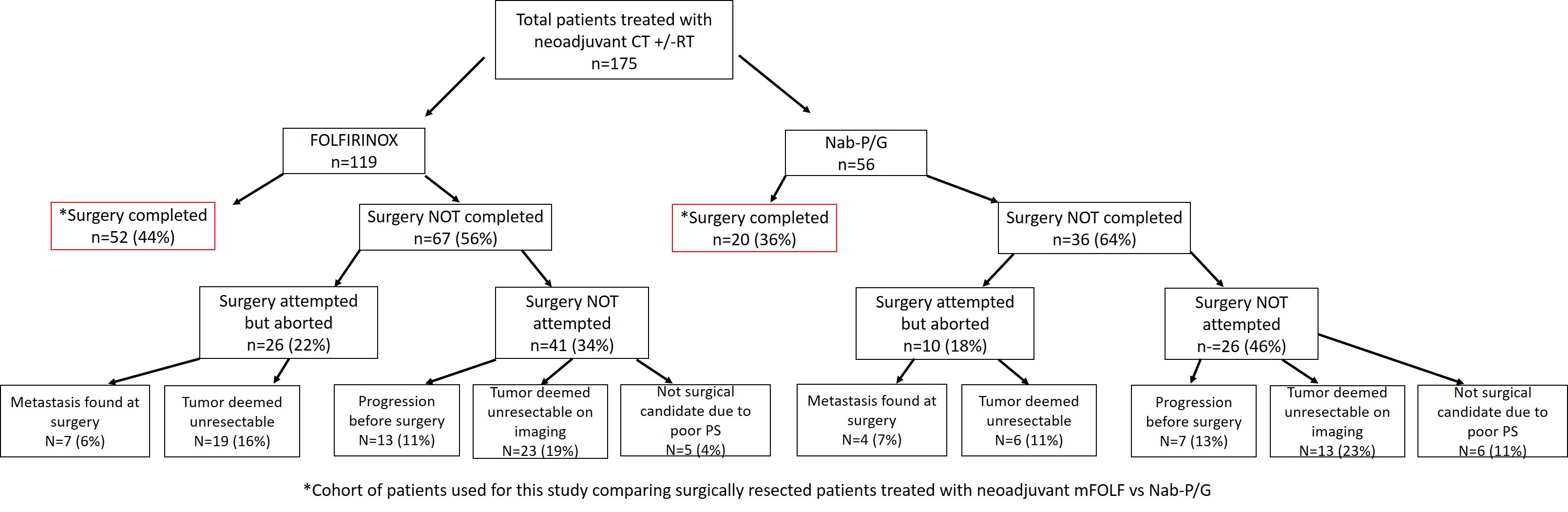

Supplement: Supplementary file 1 — Fig S1 [file CAM4-9-4711-s001.jpg]

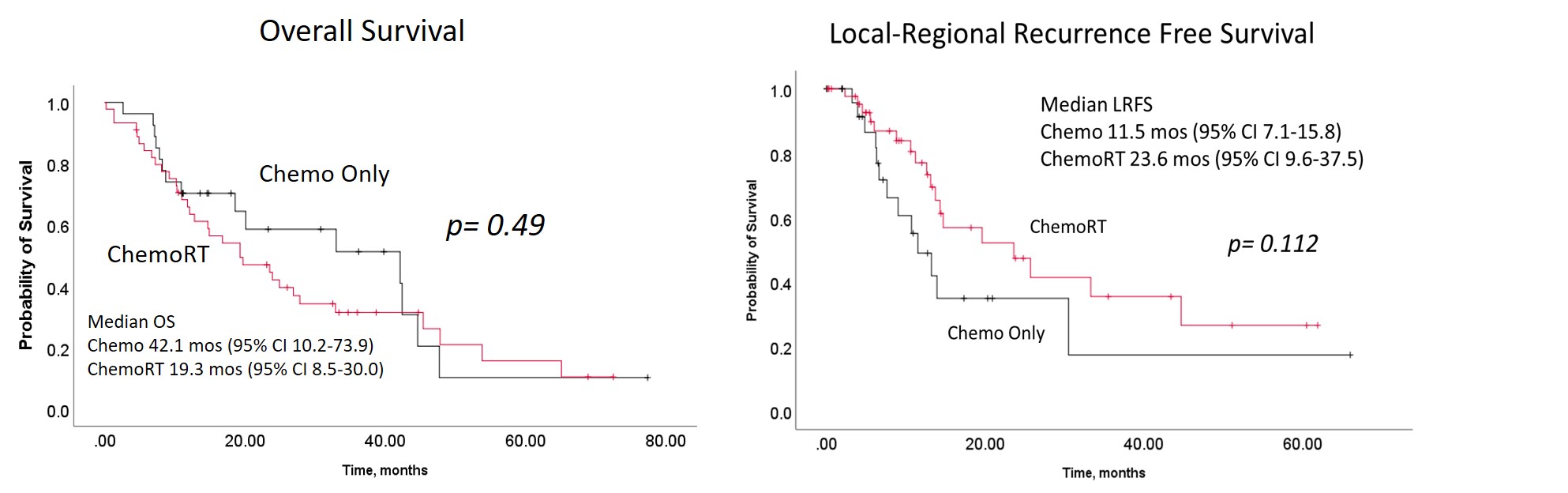

Supplement: Supplementary file 2 — Fig S2 [file CAM4-9-4711-s002.jpg]

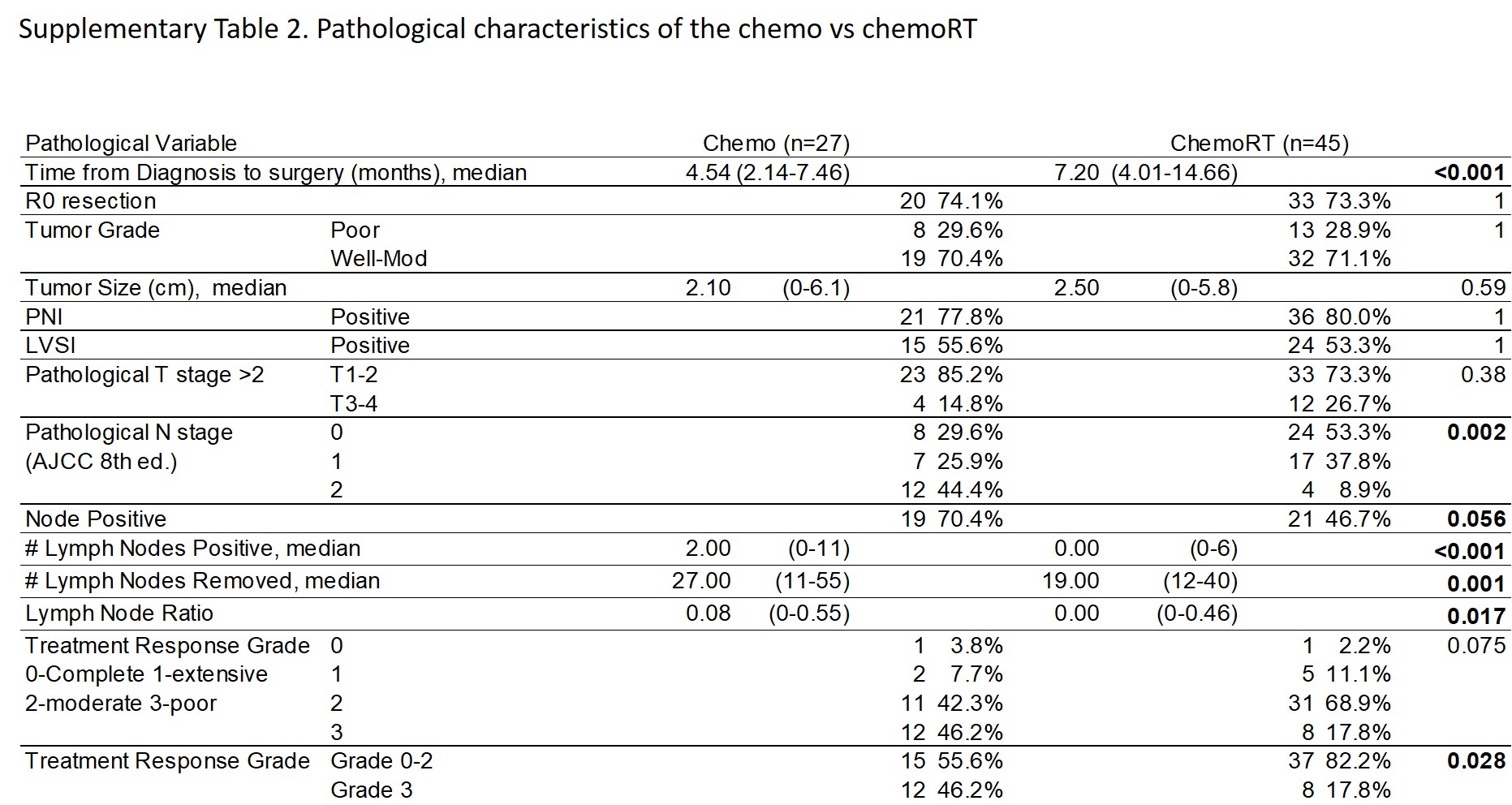

Supplement: Supplementary file 4 — Table S2 [file CAM4-9-4711-s004.jpg]
