# Supplementary material for: Neoadjuvant‐modified FOLFIRINOX vs nab‐paclitaxel plus gemcitabine for borderline resectable or locally advanced pancreatic cancer patients who achieved surgical resection
Source: Cancer Med. 2020 May 16;9(13):4711–23. doi: 10.1002/cam4.3075 (PMC7333854; doi:10.1002/cam4.3075)
Supplement: Supplementary file 3 — Table S1 [file CAM4-9-4711-s003.docx]

Supplementary Table 1. Univariate Hazard Ratios for OS and DMFS.

| **Univariate Cox HR Analysis** |  | **OS** | | | **DMFS** | | |
| --- | --- | --- | --- | --- | --- | --- | --- |
|  |  | **p* value | HR | 95% CI | **p* value | HR | 95% CI |
| Clinical variables |  |  |  |  |  |  |  |
|  | Age, continuous | **0.018** | 1.044 | 1.008-1.083 | 0.731 | 1.006 | 0.970-1.044 |
|  | Race, white vs black | 0.342 | 0.419 | 0.069-2.522 | 0.189 | 1.453 | 0.832-2.536 |
|  | Sex, male vs female | 0.813 | 0.965 | 0.722-1.292 | 0.38 | 0.75 | 0.394-1.426 |
|  | ECOG >0 vs ECOG 0 | 0.143 | 1.241 | 0.93-1.657 | 0.77 | 1.079 | 0.649-1.793 |
|  | BMI (kg/m^2) | 0.426 | 1.02 | 0.971-1.072 | 0.772 | 0.991 | 0.935-1.051 |
|  | Smoker, yes vs no | 0.762 | 1.045 | 0.785-1.391 | 0.634 | 0.856 | 0.451-1.623 |
|  | Diabetes, yes ns no | 0.128 | 0.781 | 0.568-1.074 | 0.094 | 0.554 | 0.277-1.105 |
|  | Resectability, BR vs LA | 0.751 | 0.953 | 0.709-1.282 | 0.85 | 0.937 | 0.478-1.836 |
|  | Tumor size pre-NAT, continuous | 0.628 | 1.065 | 0.825-1.376 | 0.371 | 1.135 | 0.860-1.497 |
|  | Clinical T-stage, 3-4 vs 1-2 | 0.735 | 0.949 | 0.699-1.287 | 0.314 | 1.186 | 0.850-1.655 |
|  | Clinical Node, Positive vs Negative | 0.987 | 0.997 | 0.722-1.378 | **0.022** | 2.21 | 1.123-4.347 |
| Treatment variables |  |  |  |  |  |  |  |
|  | NAT Chemo, Nab-P/G vs mFOLF | 0.109 | 0.764 | 0.55-1.062 | **0.047** | 0.477 | 0.229-0.990 |
|  | NAT RT, yes vs no | 0.675 | 0.936 | 0.689-1.273 | 0.906 | 0.96 | 0.482-1.908 |
|  | Induction Chemo Cycles, continuous | 0.72 | 0.962 | 0.776-1.191 | 0.448 | 0.912 | 0.720-1.156 |
|  | Total Radiation Dose (Gy), continuous | 0.399 | 1.024 | 0.969-1.081 | 0.576 | 1.018 | 0.957-1.082 |
|  | Time from Diagnosis to surgery (months), continuous | 0.594 | 1.032 | 0.918-1.161 | 0.409 | 1.056 | 0.928-1.201 |
| Treatment Response variables |  |  |  |  |  |  |  |
|  | Ca-19-9, Normal vs Abnormal Pre-NAT | 0.231 | 1.263 | 0.862-1.85 | **0.032** | 3.13 | 1.104-8.879 |
|  | Ca-19-9, Normal vs Abnormal Post-NAT | 0.256 | 1.181 | 0.887-1.573 | **0.013** | 2.283 | 1.194-4.365 |
|  | Ca-19-9, Normal vs Abnormal Post-Surgery | **0.035** | 1.863 | 1.045-3.319 | **0.001** | 3.021 | 1.535-5.947 |
|  | RECIST Response, PD+SD vs PR+CR | 0.291 | 0.838 | 0.603-1.164 | 0.951 | 1.022 | 0.506-2.064 |
|  | Change in Tumor Size Pre-NAT vs Post-NAT (cm), continuous | 0.307 | 0.994 | 0.983-1.005 | 0.985 | 1.0 | 0.986-1.014 |
| Pathological Variables |  |  |  |  |  |  |  |
|  | R0 vs R1 resection | **0.001** | 2.966 | 1.565-5.622 | **0.036** | 2.102 | 1.050-4.206 |
|  | Tumor Size, continuous | **0.051** | 1.206 | 1-1.456 | **0.016** | 1.299 | 1.051-1.606 |
|  | Tumor Grade, Well-Mod vs Poor | 0.652 | 1.072 | 0.793-1.448 | **0.009** | 1.91 | 1.175-3.105 |
|  | PNI, positive vs negative | 0.116 | 1.989 | 0.845-4.685 | 0.241 | 1.688 | 0.703-4.051 |
|  | LVSI, positive vs negative | 0.853 | 1.055 | 0.597-1.864 | 0.973 | 1.011 | 0.534-1.916 |
|  | Pathological T stage | **0.09** | 0.752 | 0.542-1.045 | **0.018** | 1.502 | 1.074-2.101 |
|  | Pathological N stage | 0.718 | 1.069 | 0.743-1.539 | 0.387 | 1.203 | 0.791-1.831 |
|  | LN(s) Positive, continuous | 0.727 | 1.021 | 0.909-1.146 | 0.125 | 1.101 | 0.974-1.245 |
|  | LN Ratio, continuous | 0.482 | 0.382 | 0.026-5.576 | 0.311 | 4.12 | 0.266-63.71 |
|  | Treatment Response Grade 2-3 vs 0-1 | **0.081** | 0.63 | 0.376-1.058 | **0.051** | 0.306 | 0.093-1.004 |

Abbreviations: ECOG- Eastern Cooperative Oncology Group, BMI-Body Mass Index, BR-borderline, LA-locally advanced, NAT-neoadjuvant therapy, RT-radiation therapy, PNI-perineural invasion, LVSI-lymphovascular invasion, LN-lymph node

*p values<0.10 were included in the multivariate analysis
